# Quasispecies variant of pre-S/S gene in HBV-related hepatocellular carcinoma with HBs antigen positive and occult infection

**DOI:** 10.1186/s13027-018-0179-4

**Published:** 2018-02-02

**Authors:** Yuri Hatazawa, Yoshihiko Yano, Rina Okada, Toshihito Tanahashi, Hiroki Hayashi, Hirotaka Hirano, Akihiro Minami, Yuki Kawano, Motofumi Tanaka, Takumi Fukumoto, Yoshiki Murakami, Masaru Yoshida, Yoshitake Hayashi

**Affiliations:** 1 Division of Gastroenterology, Department of Internal Medicine, Kobe University Graduate School of Medicine, Kobe, Hyogo 650-0017 Japan; 2 Division of Molecular Medicine & Medical Genetics, Department of Pathology, Kobe University Graduate School of Medicine, Kobe, Japan; 3 Department of Internal Medicine, Tokushima Prefectural Naruto Hospital, Tokushima, Japan; 4 Division of Hepato-Biliary-Pancreatic Surgery, Department of Surgery, Kobe University Graduate School of Medicine, Kobe, Japan; 5 Department of Hepatology, Osaka City University Graduate School of Medicine, Osaka, Japan

**Keywords:** HBV, Pre-S/S, Quasispecies, Occult, HCC, Ultra-deep sequencing

## Abstract

**Background:**

Hepatocellular carcinoma (HCC) can develop in patients who are negative for the hepatitis B surface antigen (HBsAg) in serum but positive for hepatitis B virus (HBV) DNA in the liver, referred to as occult HBV infection (OBI). Previous reports showed that HBV variants in OBI-related HCC are different from those in HBsAg-positive HCC. In the present study, HBV quasispecies based on the pre-S/S gene in OBI-related HCC patients were examined by high throughput sequencing and compared with those in HBsAg-positive HCC.

**Methods:**

Nineteen tissue samples (9 OBI-related and 10 HBsAg-positive non-cancerous tissues) were collected at the time of surgery at Kobe University Hospital. The quasispecies with more than 1% variation in the pre-S/S region were isolated and analysed by ultra-deep sequencing.

**Results:**

There were no significant differences in the major HBV populations, which exhibit more than 20% variation within the entire pre-S/S region, between OBI-related HCC and HBsAg-positive HCC. However, the prevalences of major populations with pre-S2 region mutations and of minor populations with polymerized human serum albumin-binding domain mutations were significantly higher in OBI-related HCC than in HBsAg-positive HCC. Moreover, the major variant populations associated with the B-cell epitope, located within the pre-S1 region, and the a determinant domain, located in the S region, were detected frequently in HBsAg-positive HCC. The minor populations of variants harbouring the W4R, L30S, Q118R/Stop, N123D and S124F/P mutations in the pre-S region and the L21F/S and L42F/S mutations in the S region were detected more frequently in OBI-related HCC than in HBsAg-positive HCC.

**Conclusions:**

Ultra-deep sequencing revealed that the B-cell epitope domain in the pre-S1 region and alpha determinant domain in the S region were variable in HBsAg-positive HCC, although the quasispecies associated with the pre-S2 region were highly prevalent in OBI-related HCC.

**Trial registration:**

Ref: R000034382/UMIN000030113; Retrospectively registered 25 November 2017.

## Background

It is estimated that approximately 248 million people worldwide are infected with the hepatitis B virus (HBV), with the highest prevalence occurring in Asia and Africa [1]. The clinical courses of chronic hepatitis B vary. Whereas some patients may progress from chronic hepatitis to cirrhosis and hepatocellular carcinoma (HCC) after HBV infection, others may naturally eliminate HBV from the blood and become hepatitis B surface antigen (HBsAg) negative [2, 3]. HBsAg is used as a marker to screen for HBV infection, and the HBsAg titre generally decreases with long-term immune responses in the host. The annual seroclearance rate of HBsAg is 0.7%–1.2% [4–6]. Recently, the goal of chronic hepatitis B therapy has been to achieve seroclearance of HBsAg [7]. However, once HBV infects the host, it integrates into the host genome and establishes a latent infection in the liver for life [8].

A serologically negative HBsAg but positive HBV DNA status in serum or the liver is referred to as occult HBV infection (OBI) [9, 10]. OBI is a potential risk factor for cirrhosis and HCC. Although it has been reported that HBV variants of the pre-S/S region are related to HCC generation and HBsAg seroclearance [11–13], the cause of carcinogenesis in OBI patients remains unclear.

The variation among viral quasispecies is conventionally analysed by direct sequencing. However, it is difficult to detect variation levels less than 20% using this method. The analysis of drug-resistance and disease-specific mutations in viruses has recently become possible following technical advances in next-generation sequencing [14, 15]. In particular, thousands of viral quasispecies within a single host can be analysed by ultra-deep sequencing methods, and it has been suggested that the detection of minor quasispecies populations are related to the clinical efficacy [16, 17].

This study focused on quasispecies based on the HBV pre-S/S gene in surgically resected HCC specimens. Using ultra-deep sequencing methods, the quasispecies were analysed and compared between OBI-related HCC and HBsAg-positive HCC. We investigated the specific mutations or variation patterns in OBI-related HCC tissues.

## Methods

### Patients and sample collection

Nineteen HCC patients who underwent surgical resection at Kobe University Hospital were enrolled in this study. Of the 19 patients, 9 (age 69.7 ± 5.6 years, 8 males and 1 female) were serologically negative for HBsAg, while 10 (age 53.0 ± 11.5 years, 9 males and 1 female) were positive for HBsAg. Laboratory data including platelet counts, transaminase levels, haemoglobin A1c, hyaluronic acid and quantitative HBsAg levels (HBsAg-HQ: Fujirebio, Tokyo, Japan) at the time of surgery were retrieved from patients’ medical records. In addition, hepatitis B core antibody (anti-HBc; Architect AUSAB, Abbott Japan, Tokyo, Japan) and HBV DNA levels (Cobas® TaqMan HBV Test, v2.0, Roche Diagnostics, Basel, Switzerland) were also examined. The cut-off levels of HBsAg-HQ and HBV DNA were estimated 5 mIU/ml and 2.06 log copies/ml, respectively. Patients negative for anti-HBc or positive for hepatitis C virus antibodies were excluded from this study.

### HBV extraction and ultra-deep sequencing analysis

Viral DNA was extracted from 200 μg of non-cancer tissue using the QIAGEN DNA Mini Kit (Qiagen, Tokyo, Japan) according to the manufacturer’s protocol. The pre-S/S region was amplified by nested PCR using specific primers [18]. The presence of an amplified product was confirmed by electrophoresis on 2% agarose gels, which were stained with ethidium bromide and visualized on a UV transilluminator. All of the necessary precautions were taken to prevent cross-contamination. A negative control was included in each assay.

Ultra-deep sequencing was used to detect the variations in HBV quasispecies. The concentrations of the PCR products were measured using the Qubit high-sensitivity double-stranded DNA assay (Invitrogen, Carlsbad, CA, USA). Next, a library of the viral genomic PCR products (< 500 bp; 50 or 0.2 ng) was prepared using the Nextera DNA sample prep kit (Illumina, San Diego, CA, USA) or Nextera XT DNA sample prep kit (Illumina), according to the manufacturer’s instructions. The PCR products were uniformly sheared to 500 bp fragments using these kits, and the PCR products were mixed with 1% 8 pM PhiX as a control and run on the MiSeq sequencer (Genome Analyzer; Illumina) for paired-end 151 bp sequencing. Finally, image-based detection of the fluorescent signals was performed using MiSeq control software (Illumina), and the images were used to obtain the sequence data in FASTQ format. Illumina paired-end sequencing was performed to generate overlapping read pairs among relatively short sequences combined with relatively long sequences. A quality check and data trimming were necessary before assembling the sequences using Genomics Workbench software version 6.0.1 (CLC bio, Aarhus, Denmark). The sequencing results used in this analysis had a read quality of > 80% of the consensus sequence estimated from a quality score of 30 according to the manufacturer’s (Illumina) data. Overlapping read pairs and filtering of reads based on a quality score of 30 were used to reveal sequencing errors and eliminate any false-positive variants generated by PCR errors during sequencing, to increase the confidence that the sequence reads can detect viral variants of low abundance.

This study was approved by the Ethical Review Board of Kobe University.

### Statistical analysis

Statistical analyses were performed using SPSS version 24.0 (IBM Corporation, S&I, Tokyo, Japan). Differences between groups were examined by Student’s *t*-test or analysis of variance. *P* values < 0.05 were considered statistically significant.

## Results

### Clinical characteristics

The average age of the OBI-related HCC patients was significantly higher than that of the HBsAg-positive HCC patients (67.7 ± 5.6 vs. 53.6 ± 11.5 years, respectively). There was no statistical significance of platelet count and transaminase levels between two groups. Among the OBI-related HCC patients, serum HBV DNA levels were undetectable, but the anti-HBc titre was 10.1 ± 1.2 (range 8.56–12.27) S/CO. Regarding the staging of liver fibrosis, no difference was found in the hyaluronic acid level and the proportion of histologically F3 (pre-cirrhosis) and F4 (cirrhosis) patients at the time of surgery between the groups (OBI 56% vs. HBsAg-positive 60%) (Table 1).

**Table 1:** Clinical and virological characteristics of the patients

	HBsAg-positive HCC (*n* = 10)	OBI-related HCC (*n* = 9)	*P* value
Age (years)	53.6 ± 11.5	69.7 ± 5.6	0.002*
Sex (male/female)	9/1	8/1	0.737
PLT (× 10^4^/mm3)	17.9 ± 4.9	23.0 ± 8.4	0.131
AST (U/L)	59.1 ± 37.9	38.9 ± 15.0	0.145
ALT (U/L)	33.2 ± 11.0	31.4 ± 15.4	0.782
HbA1c (%)	5.29 ± 0.45	5.46 ± 0.50	0.461
HBsAg (mIU/ml)	3278 ± 3045	0	
Anti-HBc antibody (C.O.I.)	12.6 ± 1.8	10.1 ± 1.2	0.002*
HBV DNA (log copies/ml)	5.41 ± 1.84	< 2.06	
Hyaluronic acid (ng/ml)	62.7 ± 51.0	107.1 ± 123.2	0.338
Fibrosis (F0–2/F3–4)	4/6	4/5	0.605
Total reads (mean ± SD)	4,451,542 ± 2,357,824	3,708,797 ± 2,365,839	0.503
Mapping reads (mean ± SD)	1,075,698 ± 845,602	619,866 ± 347,934	0.144
Coverage (mean ± SD)	131,921 ± 97,014	77,279 ± 49,215	0.139

*P < 0.05

### Ultra-deep sequencing analysis

The numbers of mapped reads and average coverage of the pre-S/S region were 859,778 ± 682,646 (range 270,715–2,941,784) and 106,038 ± 81,043 (range 30,487–301,730), respectively. The amino acids (aa) accounting for more than 1% of the variation comprised 55.1% of the aa in the pre-S/S domain, with no significant difference between the OBI and HBsAg-positive groups (Table 2).

**Table 2:** Amino acid mutations in the pre-S/S region of the major quasispecies populations

Domain	AA Position (number)	HBsAg Positive (%)	OBI (%)	P
PreS1	aa1–119 (119)	2.80 ± 1.69	4.22 ± 3.31	0.12
NTCP	aa2–48 (47)	0.60 ± 0.84	0.33 ± 0.50	0.21
HSP70	aa74–118 (45)	0.60 ± 0.70	0.33 ± 0.50	0.18
S promoter	aa66–111 (46)	0.70 ± 0.67	0.33 ± 0.50	0.10
NBS	aa103–127 (25)	0.20 ± 0.63	0.56 ± 0.88	0.16
T cell epitope	aa21–30,52–67 (26)	0.50 ± 0.71	0.67 ± 0.87	0.33
B cell epitope	aa12–47,72–78,94–117 (67)	0.70 ± 0.82	0.00 ± 0.00	0.01*
S	aa1-227 (227)	8.10 ± 8.62	7.33 ± 4.74	0.41
Pre-a determinant	aa100–119 (20)	1.20 ± 0.79	1.11 ± 0.33	0.38
a determinant	aa 124–148 (25)	0.70 ± 0.82	0.00 ± 0.00	0.01*
Post a determinant	aa 149–169 (21)	0.30 ± 0.67	0.22 ± 0.67	0.40
HLA class I	aa87–98, 186–197, 215–223 (29)	1.80 ± 1.81	1.44 ± 0.73	0.29
HLA class II	aa97–106, 171–179, 206–215 (29)	0.50 ± 0.85	0.44 ± 0.73	0.44

*P < 0.05

The consensus sequence was determined by the NCBI Blast program (available at https://blast.ncbi.nlm.nih.gov/Blast.cgi) using the sequences obtained in this study. Based on the phylogenetic analysis, all of our strains were classified into genotype C2. The closest reference HBV genotype was C2 (GenBank accession no. AB014394). The viral quasispecies were evaluated in terms of their prevalence in the viral population using the setting “read conflict” in Genomics Workbench. Based on the nucleotide alterations detected, the viruses with aa changes were determined to be variants. The proportion of a variant population was determined as the percentage of aa substitutions per aa coverage depth (entire number of sequence reads at each position) [19].

The aim of this study was to reflect the quasispecies variants in relation to hepatocarcinogenesis; therefore, we analysed variants detected in > 1% of the total viral population. A similar cut-off was applied in previous studies [20, 21]. In addition, > 20% of the total viral population is possible to detect the conventional sequencing method. In this reason, the viral quasispecies were divided and analysed; viral quasispecies detected in > 1% of the total viral population were classified as a major population (20–80% of the total population) and a minor population was defined as that comprising > 1% to < 20% of the total population, respectively.

### Amino acid variations in the pre-S region

**Fig. 1:**
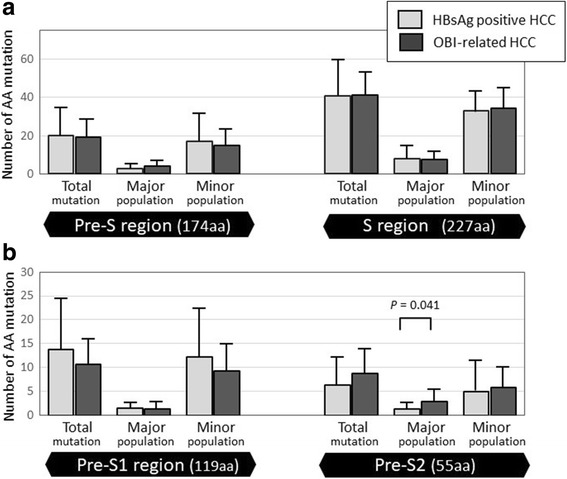
Number of aa mutations in the pre-S/S region. Number of aa mutations in the pre-S/S region (**a**), and mutations were also shown in pre-S1 and pre-S2 region (**b**). No significant difference in the average number of aa mutations was detected between HBsAg-positive and OBI-related HCC. The proportion of the major variant populations based on the pre-S2 region was significantly higher in OBI-related HCC than in HBsAg-positive HCC

Within the 174 aa pre-S region, the number of major variant populations detected were 4.22 ± 3.31 in OBI-related HCC and 2.80 ± 1.69 in HBsAg-positive HCC, respectively. Although no statistical difference in the proportion of the major quasispecies populations in the entire pre-S or pre-S1 regions was detected, the proportion in the pre-S2 region was significantly higher in OBI-related HCC than in HBsAg-positive HCC (2.89 ± 2.32% vs. 1.30 ± 0.95%, *P* = 0.041) (Fig. 1).

According to the various domains of the pre-S region, major HBV populations based on aa mutations in the B-cell epitope within the pre-S1 region were detected only in HBsAg-positive HCC (*P* = 0.01) (Table 2). On the other hand, the number of minor populations based on mutations in the polymerized human serum albumin (pHSA) domain within the pre-S2 region was significantly higher in OBI-related HCC compared with HBsAg-positive HCC (2.44 ± 2.30 vs. 0.80 ± 1.48, *P* = 0.039). The different mutations detected were W4R, S5A/P/T, L30S, G35R, H51P/R, E54K, A60V, W77R/Stop, I84T, N98 K/T, G102R, Q118R/Stop, N123D and S124F/P. Among them, the W4R, L30S and Q118R/Stop mutations were more prevalent in OBI-related HCC (Table 3).

**Table 3:** The frequencies of specific amino acid mutations associated with the major and minor population of HBV quasispecies

Region	Position	HBsAg positive (n = 10)	OBI (*n* = 9)	*P* value
Major population				
S	I126S/T	30	0	0.040
	**Y200F/S**	**0**	**33.3**	**0.025**
	Y206C/H/S	30	0	0.040
Minor population				
Pre-S	**W4R**	**0**	**33.3**	**0.025**
	S5A/P/T	30	0	0.040
	**L30S**	**10**	**77.8**	**0.001**
	G35R	30	0	0.040
	H51P/R	30	0	0.040
	E54K	50	0	0.006
	A60V	30	0	0.040
	W77R/Stop	30	0	0.040
	I84T	60	11.1	0.014
	N98 K/T	30	0	0.040
	G102R	30	0	0.040
	**Q118R/Stop**	**0**	**44.4**	**0.008**
	**N123D**	**10**	**44.4**	**0.049**
	**S124F/P**	**0**	**33.3**	**0.025**
S	L21F/S	70	100	0.040
	**L42F/S**	**0**	**33.3**	**0.025**
	W182Stop	30	0	0.040
	L213F/I/T	40	0	0.017

Bold indicated the amino acid position which mutational frequencies in OBI were higher than those in HBsAg positive

### Amino acid variations in the S region

In the 227 aa S domain, major HBV quasispecies populations were detected in 7.33 ± 4.74 of OBI-related HCC and 8.10 ± 8.62 of HBsAg-positive HCC, but there was no significant difference between the groups (Fig. 1). Major populations based on the alpha determinant region (aa 124–148, 25 aa) were detected in HBsAg-positive HCC (*P* = 0.01) but not in OBI-related HCC (Table 2). Differences in the major populations were attributed to the I126S/T, Y200F/S, and Y206C/H/S mutations, while those in the minor populations were attributed to the L21F/S, L42F/S, W182Stop, and L213F/I/T mutations. I126S/T, known as an escape mutation in the alpha determinant region, was found only in HBsAg-positive HCC (Table 3).

## Discussion

Several epidemiological and molecular studies have reported OBI as a risk factor for cirrhosis and HCC [10, 22–25]. The detection of OBI is variable in depend on the HBsAg detection kit. In particular, several older HBsAg detection kits cannot detect escape mutations in the alpha determinant region. More recently, improvements in HBsAg detection kits have made it possible to detect HBsAg, including escape mutants. The sensitivity of HBsAg detection is also improving, and most of the available kits can detect very low levels of HBsAg. The Lumipulse HBsAg HQ assay, which was used in this study, can detect extremely low levels of HBsAg, with a cut-off level of > 0.0005 IU/ml [26]. In this study, all serum HBV DNA levels were below the sensitivity threshold but HBV DNA was detected in the liver tissues. OBI was clearly confirmed, even when using a highly sensitive HBsAg kit.

All patients who were negative for HBsAg but positive for anti-HBc were treated in this study. This condition is generally considered seropositive OBI, and is treated separately from anti-HBc- and anti-HBs-seronegative OBI [9, 10]. Seropositive OBI is considered to indicate the resolved phase of acute hepatitis or the recovery phase of chronic hepatitis. Chu and Liaw reported that the presence of cirrhosis is an independent predictive factor for HBsAg seroclearance [4]. In this study, all patients showed high anti-HBc antibody titres and were thought to have had overt chronic active hepatitis. In fact, there was no difference in liver fibrosis between the OBI and HBsAg-positive cases, and five of the nine OBI cases had a histologically advanced stage (F3/4).

HBV generally exists as numerous quasispecies in the host because of the lack of a proofreading function during the reproduction process. Many studies have reported that the small number of viral variants was related to disease progression and the therapeutic response. It is thought that the small number of viral variants and the extent of variation are partly related to the clinical condition. Several reports revealed that the variation in, or deletion of, the pre-S/S region was related to the development of liver cancer as well as disappearance of the HBsAg in OBI patients. Previously, the M1L, Q2K and S182Stop in the pre-S2 region and T105C in the pre-S/S region were reported as specific mutations in OBI patients [27]. In this study, we could not detect any unique mutations in the OBI patients. So far, direct sequencing is the most common method used to detect viral mutations. However, variations present in approximately less than 20% of viral quasispecies cannot be detected by direct sequencing [28, 29]. The recent introduction of next-generation sequencing enabled analysis of viral sequence variations in more detail. Using next-generation sequencing, an increased number of reports have revealed that minor variants are related to the clinical condition [30, 31]. In this study, several mutations in the minor populations were detected in OBI patients. Further studies are necessary to clarify the clinical implications.

**Fig. 2:**
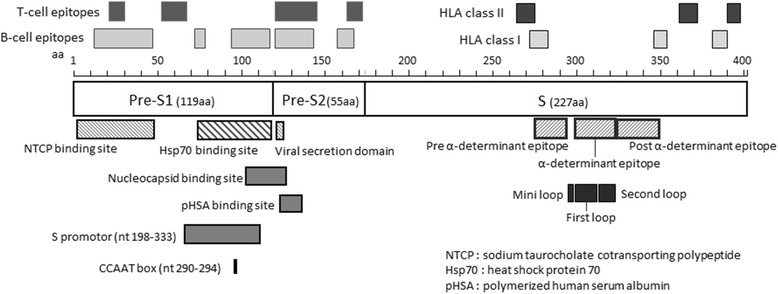
Scheme of the HBV pre-S/S region including the functional domains. NCTP-binding site in the pre-S1 region plays an important role in viral recognition and entry into hepatocytes. The alpha determinant epitope domain in the pre-S2 is involved in HBsAg production

The HBsAg is produced from the S region of HBV. The pre-S/S region contains many important domains, including the major hydrophilic region (MHR) related to the host immune response, a specific domain recognizing hepatocytes in the host, and an antigen determinant region (Fig. 2). The HBsAg becomes the target of host immunoreactivity by vaccine-induced and naturally acquired antibodies; in particular, the domain of the aa 100–169 is known as MHR, which contains the B-cell epitope and is recognized by HBs antibody. Though the detailed border of the recognition part is unknown, it has been clarified that antigen determining region α (aa124–147) is strongly related to the binding by anti-HBs antibody. In addition, it is known that the mutations and the variety in MHR domain are related to the antigenic change of the HBsAg, the evasion of the immune response from HBs antibody, the responsiveness from immune induction therapy such as globulin, and a diagnostic failure by the conventional diagnosis tool of HBsAg [32, 33]. In this study, the prevalence of the major variant population in antigen determining region α was significantly lower among OBI-related HCC than in HBsAg-positive HCC. We suggest that this region would be conserved in OBI patients because the host immune response was smaller than that in HBsAg-positive patients. Several specific mutations, including G119R, C124Y, I126S, Q129R, S136P, C139R, T140I, K141E, D144A, and G145R, have been reported as being related to the decrease of HBsAg secretion [34]. In present study, I126S/T mutants were detected only in HBsAg-positive cases, and it was suggested that the titre of HBsAg of these cases might decrease over a long-term follow-up period.

The present study revealed that the prevalence of the major variant populations associated with the B-cell epitope, within the pre-S1 region, was significantly higher in HBsAg-positive HCC than in OBI-related HCC. The greater variation in the pre-S/S region, especially the B-cell epitope, in HBsAg-positive HCC compared with OBI-related HCC, is possibly a mechanism to avoid immune pressure from the host. In contrast, the prevalence of major populations in the pre-S2 region, especially the pHSA domain, was significantly higher in OBI-related HCC than in HBsAg-positive HCC. The pre-S2 region comprises 55 aa and, together with the pre-S1 and S regions, encodes the surface proteins of HBV [35, 36]. In addition, the pre-S2 region contains the T-cell epitope, B-cell epitope, and pHSA-binding domain domains (Fig. 2). pHSA is one of the receptors that binds the HBV Dane particle [37]. The pre-S2 region is associated with HCC. It was reported that deletion of aa 1–6 of the pre-S2 region, encoding the B cell epitope, is related to HCC development [38]. In addition, a previous study reported that aa mutations in the pre-S2 region, especially the pHSA domain, are prevalent in HBV-related HCC [36], and HBV-infected patients with mutations in the pHSA region frequently progress to cirrhosis and HCC [39]. These previous studies suggested that the pre-S2 region does not play an important role in the synthesis or secretion of viral particles or in the infectiousness of the virus, but the accumulation of viral particles in hepatocytes with pHSA mutations is related to cytotoxic accumulation and progression to cirrhosis and HCC. The results of the present study suggest that a high level of variation in the pre-S2 region, particularly the pHSA domain, is related to hepatocarcinogenesis, even in OBI patients with low viral tites.

The limitations of the present study include the small number of Japanese cases evaluated. In addition, the average age of present study is different in two groups. It is usual that HBsAg positive HCC patients are younger than other cause of HCC patients. Although HBV is naturally mutated over time, it was none the less important that variation in the pre-S2 was detected in OBI patients. Future studies with larger cohorts including several viral genotypes and various host genetic factors are required to overcome these limitations.

## Conclusion

Viral quasispecies based on the pre-S/S region were less diverse in OBI-related HCC than in HBsAg-positive HCC. The most significant variation was in the B cell epitope within the pre-S1 region and the MHR domain within the S region. It is suggested that the evasion mechanism from the host immune response is weaker in OBI-related HCC. Furthermore, because mutations in the pre-S2 region were significantly prevalent in OBI-related HCC, variation in this region may be related to hepatocarcinogenesis in OBI patients.

## Abbreviations

HBV: Hepatitis B virus
HCC: Hepatocellular carcinoma
OBI: Occult HBV infection
